# Treatment of Fulminant Autoimmune Hepatitis: Corticosteroid Therapy or Liver Transplantation? A Case Report and Literature Review

**DOI:** 10.4021/gr323w

**Published:** 2011-09-20

**Authors:** Yen-Nien Lin, Jen-Wei Chou, Ken-Sheng Cheng, Cheng-Yuan Peng, Long-Bin Jeng, I-Ping Chiang

**Affiliations:** a Department of Internal Medicine, China Medical University Hospital, Taichung, Taiwan; b Division of Gastroenterology and Hepatology, Department of Internal Medicine, China Medical University Hospital, Taichung, Taiwan; c Organ transplantation center, China Medical University Hospital, Taichung, Taiwan; d Department of Pathology, China Medical University Hospital, Taichung, Taiwan; e School of Medicine, China Medical University, Taichung, Taiwan; f These authors contribute equally to this article

**Keywords:** Hepatitis, Autoimmune, Steroids, Liver failure, Acute, Liver transplantation, Plasma exchange

## Abstract

Autoimmune hepatitis initially presenting as fulminant hepatic failure is rare in clinical practice. Although corticosteroid is considered as a good therapeutic agent in treating autoimmune hepatitis in the literature, the effect of corticosteroid in treating fulminant autoimmune hepatitis is still controversial. Because corticosteroid therapy for fulminant autoimmune hepatitis can sometimes overlook any future treatment such as delay the timing of liver transplantation and precipitate postoperative complications. We report a case of a 41-year-old female who was admitted to our hosptal because of acute hepatitis with severe jaundice. Type 1 autoimmune hepatitis complicated by fulminant hepatic failure was diagnosed on the basis of her clinical course and laboratory findings. Although we prescribed aggressive medical treatment, plasma transfusion, and plasma exchange therapy, her liver function deteriorated progressively and she developed hepatic coma later. Finally, her fulminant hepatic fuilure gained dramatic improvement after receiving an orthotopic liver transplant from her younger brother. High MELD score and poor treatment response of corticosteroid therapy are indicators of poor prognosis and need of prompt OLT. Moreover, the preoperative interventions should be applied carefully ensuring that they do not delay OLT or precipitate postoperative complications such as infection, bleeding, or poor wound healing.

## Introduction

Fulminant hepatic failure is a rare complication of autoimmune hepatitis (AIH). This acute severe form of AIH can develop from de novo inflammation or by transformation from an exacerbated chronic inflammation. It usually presents with characteristic laboratory features (high serum aminotransaminase, total bilirubin, and prolonged prothrombin time) and histological findings (more frequent centrilobular zone 3 necrosis with plasmacytic infiltration and bile duct injury) [1]. Although corticosteroid therapy is effective in patients with AIH, some with fulminant hepatic failure do not respond to the therapy and eventually require orthotopic liver transplantation (OLT) [2, 3]. Herein, we describe a case of AIH presenting as fulminant hepatic failure that finally recovered after OLT. In this report, we discuss the role of corticosteroid therapy and OLT in treating fulminant AIH. In addition, we emphasize that care should be taken that any preoperative intervention should not delay OLT or precipitate postoperative complications.

## Case Presentation

**Figure 1:**
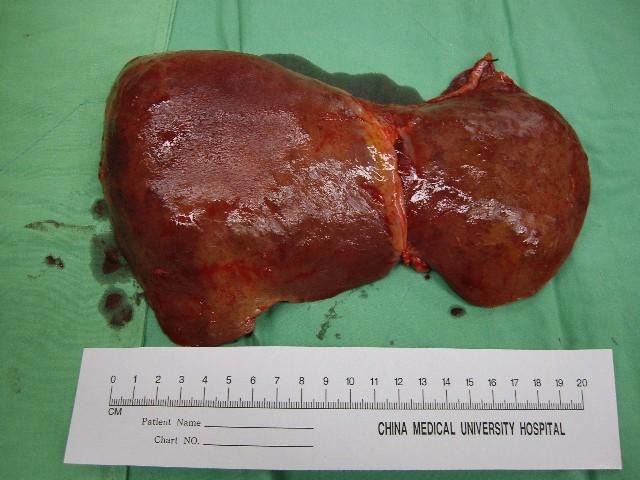
The explanted liver was shrunken, measuring 17.5 x 13.0 x 6.0 cm in size and 520 gm in weight.

**Figure 2:**
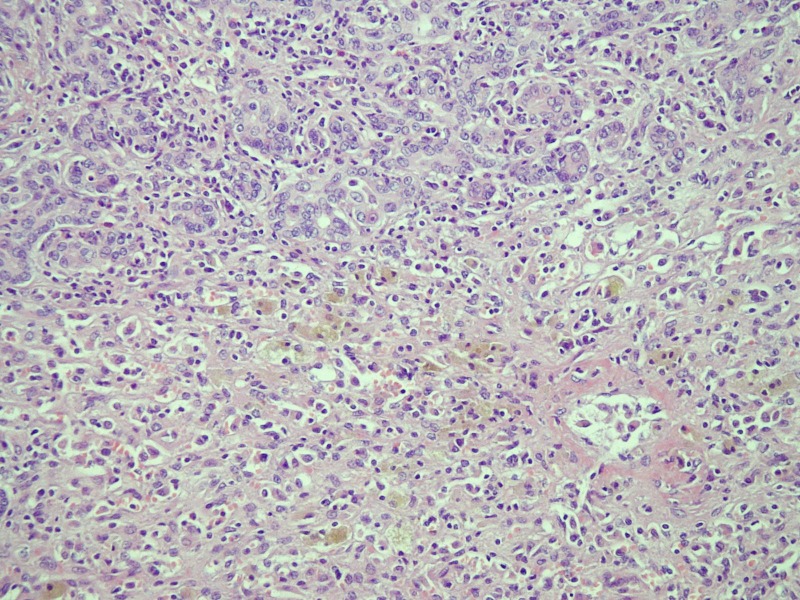
Microscopic sections of the explanted liver showed massive hepatocellular necrosis with collapse, proliferation of the bile ducts, and infiltration of numerous inflammatory cells (hematoxylin and eosin staining; x 100).

**Figure 3:**
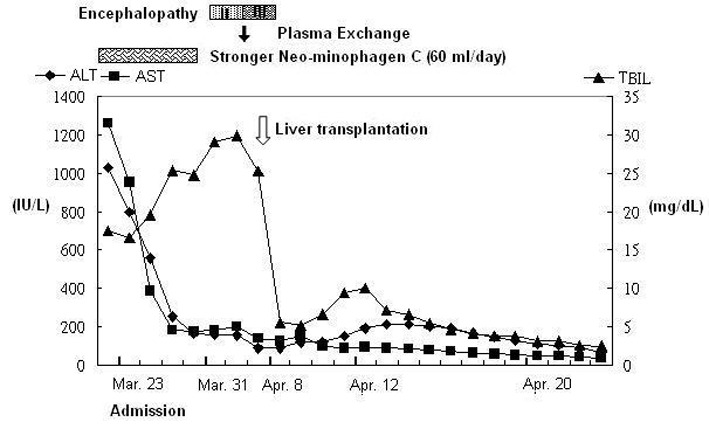
The time course of changes in the serum levels of liver enzymes and total bilirubin.

In March 2010, a 41-year-old woman visited our emergency department with a 7-day history of yellowish skin and tea-colored urine. Since a month before this presentation, she was suffering from nausea, poor appetite, malaise, and epigastralgia. She denied having fever, dyspnea, habit of alcohol consumption, and history of intravenous drug abuse or liver disease. Thereafter, she had taken some herbal medicines, but they did not improve her condition. Moreover, she lost 4 kg of weight within a month. At 7 days before admission at our department, she developed yellowish skin with general itching and tea-colored urine. On admission, physical examination revealed that her consciousness was clear. Her vital signs were as the follows: body temperature, 36.3 °C, blood pressure, 118/82 mmHg, pulse rate, 79 beats per minutes, and respiration rate, 18 breaths per minutes. Her conjunctivae were pink, and her sclerae were icteric. The results of her heart and chest examinations were normal. Her abdomen was distended with mild tenderness. There was no palpable liver or spleen. Her limbs did not show pitting edema. Laboratory test results were as the follows: serum total bilirubin, 17.4 mg/dL (reference range, 0.2-1.3), with 60% fraction of direct bilirubin; alanine aminotransferase (ALT), 1031 IU/L (0-40); aspartate aminotransferase (AST), 1261 IU/L (0-35); alkaline phosphatase, 168 IU/L (38-126); γ-glutamyltransferase, 118 IU/L (8-50); lactate dehydrogenase, 227 IU/L (98-192); albumin, 3.1 g/dL (3.5-4.9); globulin, 3.8 g/dL (2.5-3.5); and prothrombin time, 24.2 seconds with an international normalized ratio (INR) of 2.12. Serum protein electrophoresis revealed increased level of polyclonal γ-globulin (2.5 g/dL; reference range: 0.7-1.3). Serological examintions were negative for viral and metabolic makers of liver disease. The serum levels of immunological markers were as follows: immunoglobulin (Ig), G 2180 mg/dL (751-1560); IgA, 410 mg/dL (82-453); and IgM, 293 mg/dL (46-304). Moreover, anti-nuclear antibodies (ANA) were positive at a titer of 1:640 with a homogenous pattern, and 1:80 with a cytoplasmic pattern. Anti-smooth muscle antibodies (ASMA) were moderately elevated at a titer of 1:80, whereas the titer of anti-mitochondrial antibodies was 1:80. Abdominal ultrasonography showed parenchymal liver disease without cirrhotic change and ascites. On the basis of the laboratory findings, the patient was diagnosed as having acute fulminant form of type 1 AIH on hospital day 7. Although 5-day intravenous administration of stronger neo-minophagen C (SNMC) (60 mL/day) and daily transfusion of fresh frozen plasma, her hepatic failure progressed. She developed stage II hepatic encephalopathy, and her serum total bilirubin level peaked at 29.8 mg/dL on hospital day 13. Therefore, she was transferred to our intensive care unit for aggressive treatment. Plasma exchange (PE) therapy of 20 units/day and administration of intravenous hydrocortisone (150 mg/day) were started immediately on hospital day 14. The following day; however, her encephalopathy progressed to stage IV with an INR of 4.23 despite the mild decline in serum bilirubin level. Therefore, the patient’s family was informed that OLT would be required. Preoperative abdominal computed tomography scanning revealed mild atrophy of the liver and ascites. Finally, on hospital day 15, the patient received a living-donor liver transplant from her younger brother. The explanted liver was shrunken, measuring 17.5 x 13.0 x 6.0 cm in size and 520 gm in weight (Fig. 1). The explanted liver showed no cirrhotic changes or tumor mass on cutting. Histopathological examination of the explanted liver showed massive hepatocellular necrosis with infultration of numerous inflammatory cells (Fig. 2). The encephalopathy, coagulopathy, and serum bilirubin level of the patients improved dramatically after OLT. The patient was discharged uneventfully on hospital day 31, and her liver function returned to normal one month later (Fig. 3).

## Discussion

AIH is a generally progressive, chronic inflammatory liver disorder of unknown cause that fluctuates in disease activity with time. On the basis of the pattern of circulating autoantibodies, this disease can be classified into 2 main types: Type 1 (ANA and ASMA) and Type 2 [anti-liver-kidney microsome-1 antibodies (ALKM-1) and/or anti-liver cytosol antibody-1 (ALC-1)]. AIH has a broad spectrum of presentations ranging from asymptomatic to fulminant hepatic failure. Most patients with AIH show clinical features of chronic hepatitis, but after the 1990s, some patients were reported to have acute, rarely fulminant, hepatitis with jaundice and marked elevation of serum transaminases level [4]. According to previous literature, type 2 AIH is believed to be associated more frequently with acute presentation, incluidng fulminant heatic failure [5]. In recent years, type 1 AIH has also been reported to frequently present with acute symptoms [4, 6]. In our case, type 1 AIH was considered on the basis of the presence of ANA and ASMA.

Since these fulminant, acute forms of AIH responds poorly to traditional treatments and have high mortality rate, early diagnosis and close surveilliance should be over-emphasized. Diagnostic criteria of AIH were established in 1992 by the International Autoimmune Hepatitis Group (IAHG) [7]. However these criteria are fairly laborious for use in day-to-day clinical practice and not all components are routinely assessed. These criteria have a diagnostic accuracy of 90%, and have recently been simplified [8]. Several parameters have also been introduced for AIH to differentiate it from acute viral hepatitis, for which corticosteroid is contraindicated. These parameters are especially useful in the cases in which liver biopsy cannot be performed due to coagulopathy, or in which serum autoantibody study cannot be performed to confirm the diagnosis. Some of the reported parameters are as follows: gender, serum γ-globulin level, and AST/ALT ratio [9]. In our case, the patient presented with an IAHG score of 17, γ-globulin level of 2.5 g/dL, and an AST/ALT ratio of 1.22, which was suggestive of definite AIH with acute presentation that warranted immediate therapy and frequent scrutiny.

Corticosteroid therapy alone or in combination with azathioprine is established as an effective treatment that suppresses inflammatory activity in 36-100% of AIH patients [2, 3]. However, the therapeutic response reduces if the diagnosis and treatment are delayed [10]. Efforts are being made to improve the results by facilitating early diagnosis and intervention; management of the adverse effects of drugs; prevention of disease relapse; and introduction of alternative medication. In the cases of AIH presenting as fulminant hepatic failure, the role of corticosteroid remains a subject of debate. Recent researches indicated that corticosteroid therapy is of little benefit and can delay OLT [2, 3]. However, this result was criticized as the uncertainty of diagnosis of reported AIH may underestimate the corticosteroid response [11]. Czaja et al suggested a 2-week or less corticosteroid trial was beneficial, and it was also helpful in predicting the indication of OLT [12]. Because of the risk of septic complication and the high serum bilirubin level on admission, we did not prescribe corticosteroid therapy to our patient immediately and instead administered SNMC therapy. SNMC, a compound mainly composed of glycyrrhizic acid, has anti-inflammatory and anti-allergic effects. Although the precise mechanism of SNMC is not clear, Yang et al reported that SNMC can effectively protect liver against fulminant hepatic failure in animal model [13].

PE therapy is an extracorporeal blood purification technique designed for the removal of large-molecular-weight substances from the plasma. It is usually performed to allow liver regeneration or in the cases awaiting liver transplantation, especially in neurologic, immunologic, or hematologic diseases [14]. The clinical application of PE therapy in acute or chronic liver failure caused by viral hepatitis, toxins, or drugs is controversial; however, it has been reported [15]. Whether recovery in these patients is related to hepatocyte repair and regeneration is uncertain. However, the mechanisms by which PE therapy contributes to recovery remain unclear. This technology may be considered to correct coagulopathy and possibly remove various toxins from the systemic circulation. Besides its use for the treatment of AIH, PE therapy has also been used to treat other autoimmune diseases such as adult-onset Still’s disease [16]. However, it has never been used for treating the fulminant form of AIH. We attempted to perform PE therapy for treating hepatic failure in our patient, but discontinued it fearing that it might delay the time of OLT. Considering rapid proogression of encephalopathy, we prepared for OLT. The entire treatment course was uneventful, and the patient remained free of septic episodes. Her encephalopathy and laboratory findings improved after the operation and she was discharged smoothly 14 days after operation.

OLT is an essential option for patients with fulminant hepatic failure, but the optimal timing of transplantation is difficult to determine. The criteria established by King’s College Hospital are the most commonly used score system for general patients [17]. In the setting of AIH with fulminant hepatic failure, the Model of End-Stage Liver disease (MELD) scoring system was introduced, according to which those patients with a score of 12 points at presentation required liver transplantation [18]. Hyperbilirubinemia that does not improve or that worsens during the treatment course has been highly predictive of early mortality (100%) and also warrants urgent OLT [12]. Miyake et al suggested that patients whose total bilirubin levels worsen during days 8-15 after the start of therapy should be considered for OLT [2]. OLT should not be delayed if the condition of AIH patients exacerbated after 2 weeks of corticosteroid therapy or if the MELD score increased [11, 19, 20]. In our present case, the MELD score was 26 on admission. The patient received OLT on hospital day 17 with the preoperative MELD score of 36 and hepatic encephalopathy at stage IV. Histopathological examinations of the removed liver tissue revealed massive necrosis and loss of identifiable hepatic architecture. Therefore, we consider the timing for OLD is optimal in our patient beacuse the explanted liver showed massive necrosis and her encephalopathy porgessed to stage IV.

In conclusion, fulminant hepatic failure is a rare complication of AIH. Early diagnosis, excluding fulminant viral hepatitis, is beneficial for the outcome of AIH. OLT is the most essential life-saving treatment in patients with hepatic failure. The use of corticosteroid therapy or PE therapy is still controversial in treating fulminant form of AIH. High MELD score and poor treatment response of corticosteroid therapy are indicators of poor prognosis and need of prompt OLT. Moreover, the preoperative interventions should be applied carefully ensuring that they do not delay OLT or precipitate postoperative complications such as infection, bleeding, or poor wound healing.
